# Nicotinamide Phosphoribosyltransferase Knockdown Leads to Lipid Accumulation in HepG2 Cells through The SIRT1-AMPK Pathway 

**DOI:** 10.22074/cellj.2020.7013

**Published:** 2020-09-08

**Authors:** Davod Ilbeigi, Mitra Nourbakhsh, Parvin Pasalar, Reza Meshkani, Hajar Shokri Afra, Ghodratollah Panahi, Mohammad Borji, Roya Sharifi

**Affiliations:** 1. Department of Biochemistry, School of Medicine, Tehran University of Medical Sciences, Tehran, Iran; 2. Department of Biochemistry, School of Medicine, Iran University of Medical Sciences, Tehran, Iran; 3. Metabolic Disorders Research Center, Endocrinology and Metabolism Molecular, Cellular Sciences Institute, Tehran University of Medical Sciences, Tehran, Iran; 4. Department of Biochemistry, School of Medicine, Shiraz University of Medical Sciences, Shiraz, Iran; 5. Department of Medical Laboratory Sciences, School of Allied Medical sciences, Iran University of Medical Sciences, Tehran, Iran

**Keywords:** Acetyl-CoA Carboxylase, Nicotinamide Phosphoribosyltransferase, Non-Alcoholic Fatty Liver Disease, Sirtiun 1, Sterol Regulatory Element-Binding Protein-1c

## Abstract

**Objective:**

Nicotinamide phosphoribosyltransferase (NAMPT), which is responsible for biosynthesis of nicotinamide adenine dinucleotide (NAD), has a regulatory role in cellular metabolism and thus, might be implicated in non-alcoholic fatty liver disease (NAFLD). This study aimed to show how NAMPT down-regulation in liver cells influences lipid metabolism and sirtiun 1 (SIRT1), as the main NAD-dependent deacetylase enzyme.

**Materials and Methods:**

In this experimental study, HepG2 cells were transfected with NAMPT siRNA and hepatic triglyceride (TG) content and SIRT1 deacetylase activity were measured by colorimetric and fluorometric methods, respectively. Gene expression of fatty acid synthase (FAS) and sterol regulatory element-binding protein-1c (SREBP- 1c) was evaluated by real-time polymerase chain reaction (PCR). Total protein level and the phosphorylated form of acetyl-CoA carboxylase (ACC) and AMP-activated protein kinase (AMPK) were also investigated by western blotting.

**Results:**

Knockdown of NAMPT significantly promoted the accumulation of TG in HepG2 cells, accompanied by a remarkable decline in SIRT1 deacetylase activity. A significant rise in the gene expression of two key lipogenic factors, FAS and SREBP-1c was also observed. These effects were also accompanied by decreased phosphorylation of ACC and AMPK. On the other hand, treatment of transfected cells with either NAD, as the SIRT1 substrate or resveratrol, as the SIRT1 activator reversed the outcomes.

**Conclusion:**

These results demonstrated a protective role for NAMPT against NAFLD and its involvement in the regulation of de novo lipogenesis through the SIRT1/AMPK pathway.

## Introduction

It is believed that non-alcoholic fatty liver disease (NAFLD) ranks among the most common liver disorders worldwide and shows increasing incidence within the past two decades (1). NAFLD ranges from simple fat deposition (steatosis) to non-alcoholic steatohepatitis (NASH), characterized by steatosis and inflammation. NASH can lead to fibrosis, cirrhosis, and eventually hepatocellular carcinoma (HCC) (2).

Liver has a central role in lipid metabolism, and synthesis, and import of free fatty acids, as well as storing and exporting lipids and lipoproteins. In NAFLD, lipid deposition is increased because of elevated hepatic lipogenesis and increased lipid uptake. At the same time, reduced lipid removal caused by decreased β-oxidation and diminished TG export, causes a positive lipid balance in the liver leading to the progression of NAFLD (3). Obesity and its metabolic consequences such as insulin resistance and type 2 diabetes mellitus, are the most prevalent risk factors for dyslipidemia and steatosis development (4).

Approximately 26% of liver lipids are produced by de novo lipogenesis and this pathway is induced in individuals with NAFLD (5). In this pathway, acetyl-CoA carboxylase (ACC) and fatty acid synthase (FAS) are major enzymes that are controlled by important transcription factors like carbohydrate response element-binding protein (ChREBP), sterol regulatory element-binding protein-1c (SREBP-1c) and liver X receptor (LXR) (6).

Nicotinamide phosphoribosyltransferase (NAMPT), an enzyme with two intra- and extracellular forms, is a 52 kDa protein expressed in approximately all tissues/ cells (7). Extracellular NAMPT (eNAMPT), generally called visfatin, is secreted by visceral adipose tissue and functions as an adipokine and activates various cellular signaling pathways (8, 9). Intracellular form of the protein (iNAMPT) has an enzymatic activity and functions as the key enzyme in NAD biosynthesis. NAD is an essential coenzyme in many metabolic reactions and has multiple roles in cellular metabolism (10). NAMPT catalyzes the synthesis of nicotinamide mononucleotide (NMN) from nicotinamide and 5-phosphoribosyl-pyrophosphate (PRPP) (11). Nicotinamide mononucleotide adenylyltransferase (NMNAT) turns NMN into NAD (12). Some enzymes use NAD as substrate for their catalytic reactions such as the sirtuin family of protein deacetylases (13); one of them is silent information regulator 1 (SIRT1) and its inhibition has been shown to be implicated in hepatic steatosis (14). SIRT1 controls the function of AMPactivated protein kinase (AMPK), which is a central enzyme in energy homeostasis and fatty acid metabolism (15, 16). ACC phosphorylation and activity are respectively increased and repressed by AMPK phosphorylation. The subsequent decline in the synthesis of malonyl-CoA causes up-regulation of fatty acid oxidation and suppression of fatty acid synthesis, which lead to decreased levels of hepatocyte lipids (17). Moreover, glucose-induced expression of FAS is impeded by AMPK activation that hampers fatty acid biosynthesis and leads to decreased TG levels (18).

SREBP-1c is one of the main transcription factors that induces the expressions of lipogenic genes such as* FAS, ACC* and glycerol-3-phosphate acyltransferase (*GPAT*), and can affect the production of fatty acids and TG. Thus, SREBP- 1c plays a critical role in NAFLD (19). It was found that AMPK can phosphorylate SREBP-1c at Ser 372, suppress its translocation into the nucleus, and therefore repress the expression of its target genes (20). Increased expression of SIRT1 was shown to be a causative factor in SREBP-1c gene expression decline in obese mice (21).

Recent studies suggested that NAMPT is involved in lipid metabolism in the liver; however, its mechanism is still not clearly defined (19, 22). A recent study showed that inhibition of NAMPT by its chemical inhibitor, FK866, aggravated hepatic lipid accumulation and steatosis *in vivo* and *in vitro* (19). Additionally, it was reported that NAMPT is necessary for *de novo* lipid biosynthesis in prostate cancer (PCa) cells (22). NAMPT expression was also shown to be reduced in the serum and liver tissue of patients with NAFLD (23).

Given the regulatory role of SIRT1 and AMPK in fatty acid and lipid metabolism, NAMPT might be related to NAFLD by provision of NAD as the main SIRT1 substrate.

Some studies showed that circulating levels of eNAMPT/visfatin are elevated in NAFLD (24, 25). Conversely, downregulation of iNAMPT was recently reported in hepatic tissue of patients with NAFLD (23). Taken together, these studies point to the involvement of NAMPT in the pathogenesis of NAFLD; however, the associated molecular and metabolic mechanisms are not fully elucidated. In the current study, we inspected the effect of NAMPT knockdown on lipid accumulation, lipogenic factors, and SIRT1 activity in HepG2 cells.

## Materials and Methods

This experimental study was approved by the Ethics Committee of Tehran University of Medical Sciences (IR. TUMS.REC1394.2182).

### Cell culture and transfection

Human hepatoma cells (HepG2) were obtained and authenticated from Iranian Biological Resource Center (Tehran, Iran). All cell culture reagents were bought from Gibco (UK). HepG2 cells were kept at 37˚C in Dulbecco Modified Eagle medium (DMEM) containing fetal bovine serum (10%) and 100 μg/ml penicillin and streptomycin, in 5% CO_2_. Cells were transfected using polyethyleneimine (PEI, Thermo, USA) as transfection reagent. Approximately, 5×10^5^ cells were seeded in 2 ml of medium in 6-well plates for 24 hours, prior to transfection. Afterwards, the cells were incubated with serum-free medium for 6 hours and subsequently transfected with PEI alone (mock) or with either siRNA against NAMPT or its negative control (scrambled siRNA) from GenePharma (Shanghai, China). FAM-labeled control siRNA (GenePharma, Shanghai, China) was also used to monitor transfection efficiency. The siRNA was mixed with PEI at the nitrogen/phosphate (N/P) ratio of six (26). Cells were incubated with the transfection complex for 24 hours. Afterwards, the transfection medium was substituted with fresh growth medium and the incubation was continued for another 24 hours. Cells with no treatment were considered control. Knockdown of NAMPT was confirmed by realtime polymerase chain reaction (PCR) and western blotting. Resveratrol was also used to investigate the involvement of SIRT1. For this purpose, two different concentrations of resveratrol (20 and 50 μM) were examined according to the cytotoxicity test done by MTT (27, 28); eventually, because of better cell viability, 20 μM concentration was chosen for the experiments. The transfected cells were treated with resveratrol (20 μM) or NAD (1 mM) separately in fresh medium for 24 hours.

### Detection of SIRT1 deacetylase activity

To investigate the relationship between NAMPT knockdown and changes in SIRT1 activity, the effect of NAMPT knockdown by siRNA on SIRT1 deacetylase activity was assessed after transfection for 48 hours using an SIRT1 Activity Assay Kit (Fluorometric, Abcam, Cambridge, UK) following the manufacturer’s protocol. Briefly, a mixture including fluoro-substrate peptides and NAD as SIRT1 substrates was mixed with cell extracts or recombinant SIRT1 as the positive control.

Then, a microplate reader was used to measure fluorescence for 60 minutes with 1-2 minute intervals at excitation and emission wavelength of 350 and 460 nm, respectively. Fluorescence intensity relative to untreated control cells was used to express the enzyme activity.

### Oil red O staining

About 5×10^5^ HepG2 cells were seeded in 2 ml of medium in 6-well plates for 24 hours and transfected as described above. After transfection, cells were washed 3 times with phosphate buffered saline (PBS, Sigma Aldrich, Germany) and fixed with 4% formaldehyde for 1 hour. Oil Red-O staining solution (0.5% in isopropanol) was added and incubated for 15 minutes at room temperature. Finally, the cells were washed 3 times with PBS. The cells were photographed under light microscopy. Isopropanol was added to the stained lipid droplets and the absorbance was measured at 492 nm.

### Intracellular triglyceride measurement

Cells were harvested 48 hours after transfection and the intracellular TG content was measured. Pellets of cells were homogenized in 1 ml of 5% NP-40 solution. Then, slow heating was applied to the mixture to 80-100°C in a water bath for 5 minutes; afterward, the mixture was let to cool down to room temperature. Then, it was centrifuged for 2 minutes at top speed for eliminating the insoluble materials. Next, a commercial kit (Abcam, UK) was applied to determine TG content of the resulting solution. The concentration of total protein was quantified by bicinchoninic acid (BCA) method by applying Pierce protein assay kit (Thermo, USA), and TG levels were presented as μg of lipid/mg of protein.

### Real-time polymerase chain reaction

RNA extraction kit (GeneAll, South Korea) was used for total RNA isolation. Reverse transcription reaction was performed using kit for cDNA synthesis (Thermo Fisher Scientific, Waltham, USA). StepOnePlus real-time PCR System (Applied Biosystems, USA) was applied using SYBR Green PCR Master Mix (Ampliqon, Denmark) to amplify the resulting cDNA. Relative gene expression was analyzed by ∆∆Ct method and *β-actin* was used as the normalizer (29). The primer sequences are presented in Table 1.

**Table 1:** The sequences of the used primers


Primers target	Primer sequence (5´-3´)

*NAMPT*	F: GGTTCTTGGTGGAGGTTTGCTAC
	R: GAAGACGTTAATCCCAAGGCC
*SREBP-1c*	F: CACCGAGAGCAGAGATGGC
	R: AAGGAGACGAGCACCAACAG
*FAS*	F: GAGGAAGGAGGGTGTGTTT
	R: CGGGGATAGAGGTGCTGA
*β-actin*	F: TCCTTCCTGGGCATGGAGT
	R: ACTGTGTTGGCGTACAGGTC


### Western blot analysis

After transfection for 48 hours, cells were lysed by radio-immunoprecipitation assay (RIPA) buffer containing phenylmethylsulfonyl fluoride (PMSF) as the protease inhibitor. The concentration of the extracted protein was assessed using the BCA method. Electrophoresis was performed on 10% sodium dodecyl sulfate (SDS) polyacrylamide gel to separate equal amounts of total proteins; then, they were transferred into polyvinylidene difluoride (PVDF) membranes. Subsequently, blocking was carried out by incubation of membranes for 3 hours at room temperature in tris-buffered saline containing 5% skim milk and 0.1% tween-20 (TBST). Afterward, the membranes were incubated overnight at 4°C, with rabbit primary antibodies (Cell Signaling Technology, USA) against NAMPT, phospho-AMPK (Thr172), total-AMPK, phosho-ACC (Ser 79), total -ACC, and GAPDH as the loading control at the dilution of 1:1000. Horseradish peroxidase-conjugated anti-rabbit antibody (Cell Signaling Technology, Danvers, USA) at 1:5000 dilution, was used as the secondary antibody. The protein bands were visualized by exposing them to X-ray film after reaction with enhanced chemiluminescence (ECL) detection reagent. Densitometric analysis of the resulting bands was performed by ImageJ software (v1.52, NIH). In order to perform the blotting with different antibodies, the membranes were stripped, re-probed and visualized after blocking and incubating with the primary and secondary antibodies.

### Statistical analyses

Data are shown as mean ± SD of at least three separate experiments. One-way analysis of variance (ANOVA) together with Dunnett’s multiple comparison post-hoc test was used to evaluate significant differences among groups. GraphPad Prism software (version 5.04., USA) was applied for statistical analysis. A P<0.05 was considered statistically significant.

## Results

### Confirmation of transfection

**Fig.1:**
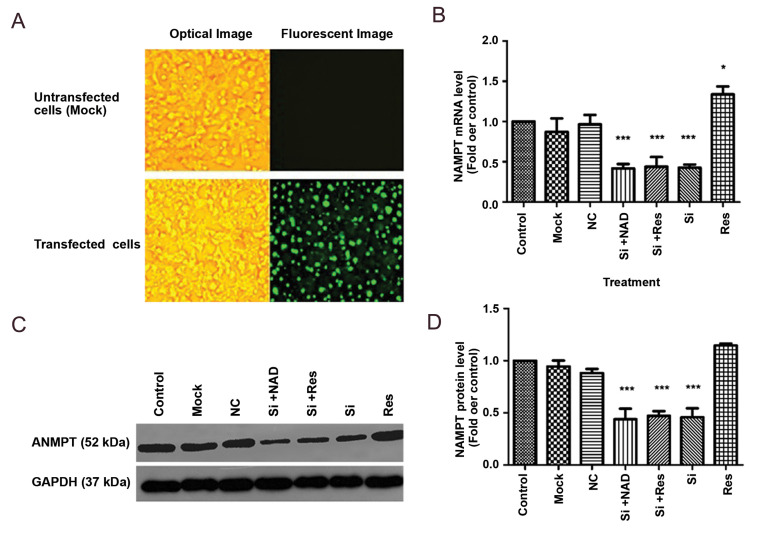
Transfection of HepG2 cells with NAMPT siRNA. **A.** Transfection efficiency was tested by FAM-siRNA under a fluorescence microscope (right panel) compared to the image under the optical microscope (left panel) (scale bar: 50 μm). **B.** Decreased expression of NAMPT mRNA measured by real-time PCR. **C.** Protein levels evaluated by western blotting compared to untreated control cells, after transfection with siRNA (Si), and **D.** A representative blotting image. Transfected cells were also treated with NAD (1 mM) and resveratrol (Res) (20 μM). A representative blotting image is shown and the data represent the mean ± SD. NAMPT; Nicotinamide phosphoribosyltransferase, PCR; Polymerase chain reaction, NAD; Nicotinamide adenine dinucleotide, *; P<0.05, ***; P<0.001 versus the control, and NC; Negative control (scrambled siRNA).

Transfection efficiency was evaluated by FAM-siRNA under a fluorescence microscope and the results were indicative of efficient transfection (Fig.1A). In addition, knockdown of NAMPT was confirmed by real-time PCR as well as western blotting. Transfection with siRNA significantly downregulated both the mRNA and protein levels of NAMPT (by about 58 and 55 %, respectively), compared to the control (P<0.001, Fig.1B, C).

### Knockdown of NAMPT reduces SIRT1 activity

**Fig.2:**
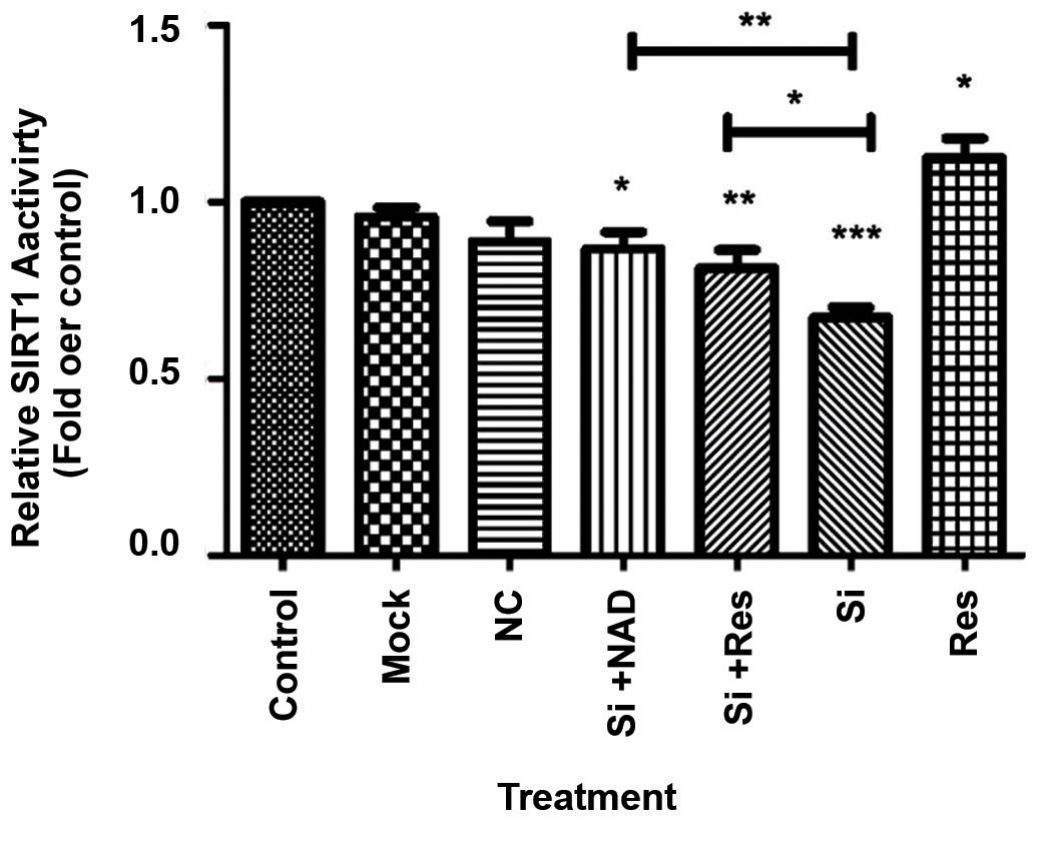
Reduced SIRT1 activity in HepG2 cells after transfection with NAMPT siRNA (Si). Resveratrol (Res) or NAD ameliorated this effect and increased SIRT1 activity. The results are mean ± SD of at least 3 independent experiments. NAMPT; Nicotinamide phosphoribosyltransferase, NAD; Nicotinamide adenine dinucleotide, *; P<0.05, **; P<0.01, ***; P<0.001 versus the control, and NC; Negative control (scrambled siRNA).

Since NAMPT provides the substrate for SIRT1 activity, we first tested the hypothesis that knockdown of NAMPT modulates SIRT1 deacetylase activity. As shown in Figure 2, when cells were transfected with siRNA, SIRT1 deacetylation activity was significantly reduced compared to the untreated control cells. This inhibition was removed when excessive amounts of NAD were provided, confirming that the reduction of SIRT1 activity was due to the loss of NAD production by NAMPT.

Resveratrol, which is a well-characterized activator of SIRT1, also significantly reversed the effect of NAMPT knockdown, further confirming that the reduced SIRT1 activity was due to the down-regulation of NAMPT.

### NAMPT affects hepatic lipid accumulation via SIRT1

**Fig.3:**
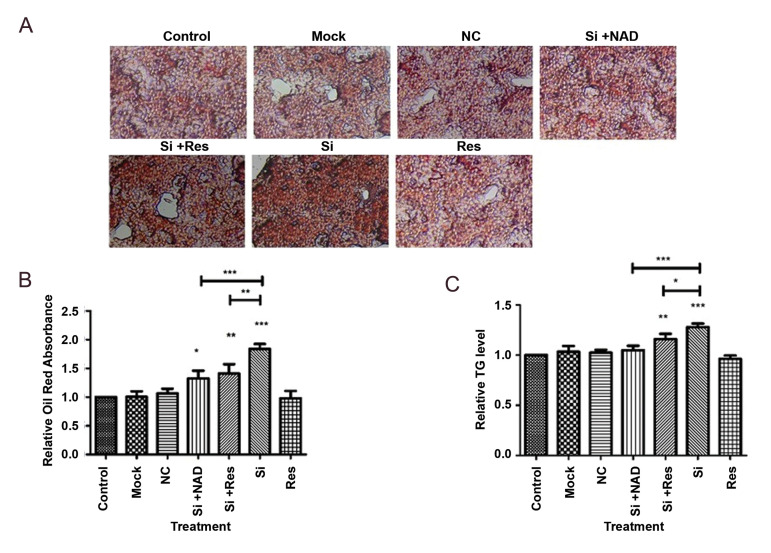
Knockdown of NAMPT with siRNA (Si) promoted TG accumulation in HepG2 cells. **A.** Microscopic images of the intracellular lipid content after Oil Red O staining (scale bar: 50 μm). **B. **Relative intracellular lipid content which was measured spectrophotometrically at 492 nm after solubilization of Oil Red O stain. **C.** Intracellular TG level determined by enzymatic method. The data represent the mean ± SD of at least three independent experiments. NAMPT; Nicotinamide phosphoribosyltransferase, TG; triglyceride, NAD; Nicotinamide adenine dinucleotide, *; P<0.05, **; P<0.01, ***; P<0.001 versus the untreated control, and NC; Negative control (scrambled siRNA).

In order to determine whether NAMPT is involved in hepatic steatosis, Oil Red O staining was performed. The results indicated that lipid content of HepG2 cells was significantly increased after NAMPT knockdown (Fig.3A, B). In addition, intracellular TG levels were measured following knockdown of NAMPT in HepG2 cells. As it is shown in Figure 3C, NAMPT knockdown caused a remarkable rise in the TG content of cells compared to the control cells. Elevation of TG levels by NAMPT knockdown was notably reversed by addition of NAD, confirming the effect of NAMPT inhibition on hepatic steatosis. Interestingly, treatment with resveratrol significantly reduced the TG accumulation that indicated the involvement of SIRT1 in the effect of NAMPT on lipid metabolism in HepG2 cells.

### NAMPT and SIRT1 cooperate in the regulation of lipid metabolism in liver cells through modulation of FAS and SREBP-1c

**Fig.4:**
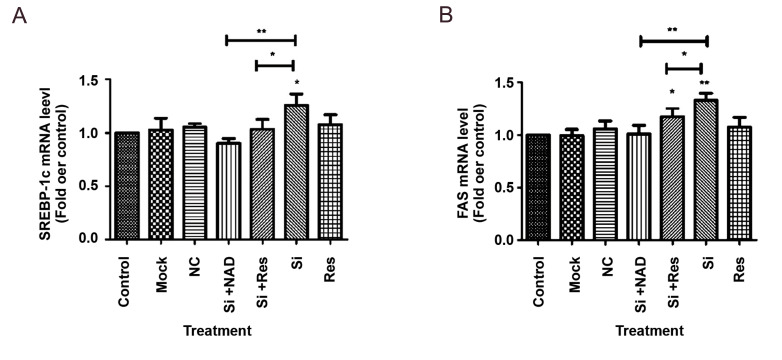
Knockdown of NAMPT with siRNA (Si) in HepG2 cells enhanced the expression of genes involved in lipogenesis. **A. **mRNA levels of *SREBP-1*, **B. **mRNA levels of *FAS*, after knockdown of NAMPT. The results are mean ± SD of at least three independent experiments. NAMPT; Nicotinamide phosphoribosyltransferase, NAD; Nicotinamide adenine dinucleotide, *; P<0.05, **; P<0.01 compared to untreated control, and NC; Negative control (scrambled siRNA).

To determine the participation of NAMPT in the gene expression of lipogenic factors in hepatocytes, HepG2 cells were transfected with NAMPT siRNA and the effect of NAMPT silencing was evaluated on *FAS* and *SREBP-1* mRNA expression. We showed that suppression of NAMPT was followed by a significant enhancement of *FAS* and *SREBP-1* expression at mRNA levels. In comparison to the untreated control cells, we achieved 28 and 30% elevation in *SREBP-1* and *FAS* mRNA levels, respectively (Fig.4A, B). The negative control siRNA had no influence on the expression of these genes. On the other hand, treatment with resveratrol or NAD reversed these effects and significantly decreased the *SREBP-1c* and *FAS* mRNA levels compared to the cells transfected with NAMPT siRNA.

### Down-regulation of NAMPT affects phosphorylation of ACC and AMPK via SIRT1

**Fig.5:**
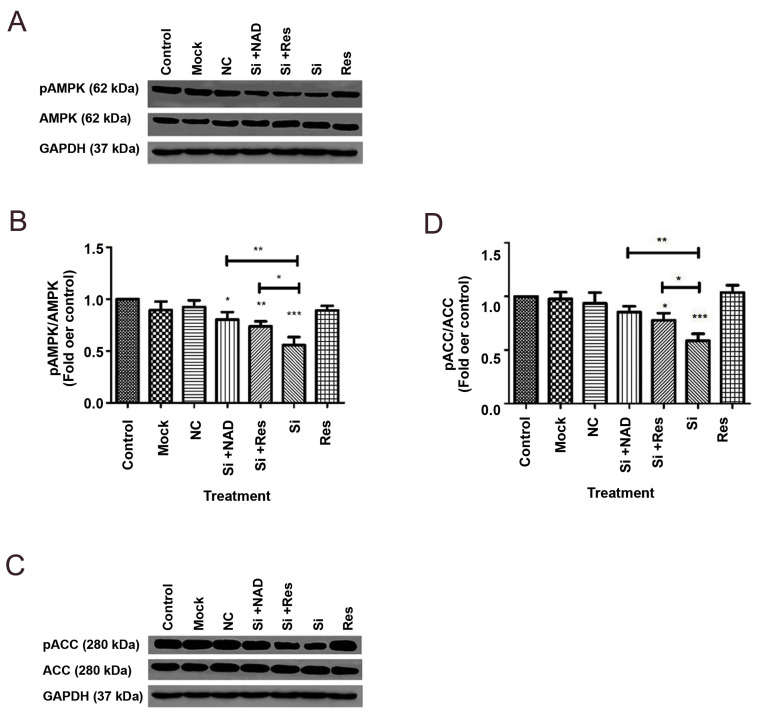
Evaluation of the effect of NAMPT knockdown with siRNA (Si) on AMPK and ACC phosphorylation in HepG2 cells by Western blotting followed by densitometric analysis of the resulting bands. **A.** A representative blotting image for pAMPK and total-AMPK. **B. **quantitative analysis of the ratio of pAMPKα to total-AMPKα. **C.** A representative blotting image for pACC and total-ACC. **D.** Quantitative analysis of the ratio of pACC to total-ACC. The levels of protein were normalized to GAPDH. The data are presented as mean ± SD of at least 3 separate experiments. *; P<0.05, **; P<0.01, ***; P<0.001 versus the untreated control, and NC; Negative control (scrambled siRNA).

To characterize whether NAMPT takes part in regulating lipid metabolism in the liver, we assessed the influence of NAMPT knockdown on AMPK and ACC as the regulatory enzymes in lipogenesis. As shown in Figure 5, down-regulation of NAMPT by siRNA caused a significant decline in the phosphorylation levels of AMPK at Thr172 and ACC at Ser 79, by 42 and 32%, respectively, compared to the control cells, while the total levels of both ACC and AMPK protein were not changed. Conversely, treatment of the transfected cells with resveratrol or NAD significantly reversed the effect of NAMPT knockdown, suggesting the requirement of NAD for AMPK phosphorylation and the involvement of SIRT1 in the activation of AMPK and inhibition of ACC.

## Discussion

NAFLD which is considered to be among the most prevalent liver disorders, is defined by the extent of lipid accumulation in liver (30). It is believed that dysregulation of lipid synthesis ranks among the major reasons causing abnormal lipid accumulation in the liver (31).

Recent studies established the involvement of NAMPT in the regulation of lipid metabolism (22, 32). In this research, we examined the effect of NAMPT knockdown by siRNA on TG accumulation, SIRT1 activity, and lipogenic factors in HepG2 liver cells. The current study presents the first direct evidence that NAMPT influences lipid metabolism in HepG2 cells and modulates TG accumulation through SIRT1/AMPK pathway.

One of the main findings of this study was that NAMPT knockdown resulted in lipid accumulation in HepG2 cells. Consistently, Wang et al. (19) and Zhou et al. (33) showed that inhibition of NAMPT aggravated hepatic lipid accumulation and steatosis. Additionally, it was reported that NAMPT is necessary for de novo lipid biosynthesis in cancer (22). These results together with our findings indicate that down-regulation of NAMPT might be an essential element contributing to the pathogenesis of NAFLD. The decreased expression of NAMPT in the hepatic tissue of animals kept on high fat diet as well as its decline in HepG2 cells treated with oleic acid is also suggestive of the role of NAMPT in controlling hepatic lipid metabolism (19, 33). NAMPT was also shown to be reduced in the liver tissue of patients with NAFLD, further confirming the relationship between NAMPT and hepatic steatosis (23). NAMPT positively regulates SIRT1 activity through the enzymatic synthesis of NAD (19). Furthermore, SIRT1 was shown to be protective against hepatic steatosis (15). The levels of SIRT1 are generally reduced in patients with NAFLD (34). On the other hand, overexpression of SIRT1 prevents high glucose-induced lipid pile-up in HepG2 cells (15). Here we showed that NAMPT knockdown decreased NADdependent deacetylase activity of SIRT1, leading to increased TG accumulation in HepG2. Supplementation of cells with NAD compensated the deleterious effect of NAMPT inhibition, pointing out the importance of NAD provision by NAMPT for the function of SIRT1.

Consistent with our results, it was reported that by reestablishing SIRT1 activity and promoting the mitochondrial effectiveness through NAD supplementation, the fatty liver is ameliorated in mice (35, 36). Furthermore, Zhou et al. (33) confirmed that the age-related NAD deficit intensified vulnerability to NAFLD and contributed to dietinduced steatohepatitis.

We also showed that treatment of transfected cells with resveratrol, a well-known and potent SIRT1 activator (37), reversed TG accumulation, confirming the involvement of SIRT1 in the effect of NAMPT on liver cells. Consistently, it was reported that treatment of 3T3-L1 adipocytes with resveratrol decreases TG accumulation and increases SIRT1 gene and protein expression (38).

Another finding of the current study was that NAMPT influenced the phosphorylation of ACC and AMPK. The phosphorylation and subsequent stimulation of AMPK lead to inhibition of ACC through phosphorylation and shift the metabolic pathways from lipogenesis to lipid oxidation. Thus, increased TG accumulation following NAMPT down-regulation, can be attributed to reduced AMPK and increased ACC activities caused by the decline in SIRT1 function.

The mRNA expression of *FAS* and *SREBP-1*, as major transcription factors in lipid metabolism, were also increased in HepG2 cells following NAMPT knockdown, an effect that was attenuated after treatment with NAD. Therefore, increased gene expression of *SREBP-1* as well as *FAS* might serve as another mechanism linking NAMPT to hepatic steatosis. In agreement with our results, Wang et al. (19) showed that NAMPT overexpression caused a dramatic decline in lipid content and decreased the expressions of genes that are responsible for the regulation of lipogenesis such as SREBP-1c, and its downstream targets including FAS and ACC.

We found that the effects of NAMPT on the above lipogenic factors were reversed by resveratrol. It was reported that SIRT1 is able to suppress the expression of FAS via activating AMPK (15). SIRT1 also downregulates hepatic SREBP-1c activity by deacetylation (21). Thus, it is suggested that the modulation of FAS and SREBP-1c by NAMPT may also occur through SIRT1.

Altogether, these results suggest a protective role for NAMPT against NAFLD. Our findings are consistent with the previous observations that serum NAMPT had a significant negative correlation with the hepatic de novo lipogenesis and liver mitochondrial function (39, 40).

## Conclusion

These data demonstrate that down-regulation of NAMPT increases hepatic lipid accumulation through modulation of SIRT1/AMPK pathway, leading to increased expression of FAS and SREBP-1c as well as reduced phosphorylation of ACC. Thus, NAMPT might be considered to be central and upstream to pathogenesis of NAFLD and regarded as a therapeutic strategy for this disorder.
